# Antimicrobial Resistance Traits of *Escherichia coli* Isolated from Dairy Manure and Freshwater Ecosystems Are Similar to One Another but Differ from Associated Clinical Isolates

**DOI:** 10.3390/microorganisms8050747

**Published:** 2020-05-16

**Authors:** Rachelle E. Beattie, Ellen Bakke, Nicholas Konopek, Rebecca Thill, Erik Munson, Krassimira R. Hristova

**Affiliations:** 1 Department of Biological Sciences, Marquette University, Milwaukee, WI 53233, USA; rachelle.bailey@marquette.edu (R.E.B.); nicholas.konopek@marquette.edu (N.K.); rebecca.thill@marquette.edu (R.T.); 2 Driskill Life Sciences, Northwestern Medicine Feinberg School of Medicine, Chicago, IL 60611, USA; ellenbakke2024@u.northwestern.edu; 3 Department of Clinical Laboratory Science, Marquette University, Milwaukee, WI 53233, USA; erik.munson@marquette.edu

**Keywords:** antimicrobial resistance, ESBL, One Health, *E. coli*, phylotyping, plasmid replicon typing, isolate association

## Abstract

Antimicrobial resistance (AMR) is a prevalent global health problem across human and veterinary medicine. The One Health approach to AMR is necessary to mitigate transmission between sources of resistance and decrease the spread of resistant bacteria among humans, animals, and the environment. Our primary goal was to identify associations in resistance traits between *Escherichia coli* isolated from clinical (*n* = 103), dairy manure (*n* = 65), and freshwater ecosystem (*n* = 64) environments within the same geographic location and timeframe. Clinical *E. coli* isolates showed the most phenotypic resistance (47.5%), followed by environmental isolates (15.6%) and manure isolates (7.7%), with the most common resistances to ampicillin, ampicillin-sulbactam, and cefotaxime antibiotics. An isolate subset was screened for extended spectrum beta-lactamase (ESBL) production resulting in the identification of 35 ESBL producers. The most common ESBL gene identified was *bla_TEM-1_*. Additionally, we found nine different plasmid replicon types including IncFIA-FIB, which were frequently associated with ESBL producer isolates. Molecular phylotyping revealed a significant portion of clinical *E. coli* were associated with phylotype B2, whereas manure and environmental isolates were more diverse. Manure and environmental isolates were significantly different from clinical isolates based on analyzed traits, suggesting more transmission occurs between these two sources in the sampled environment.

## 1. Introduction

Antimicrobial resistance (AMR) is a prevalent and persistent problem in public health across all natural and man-made environments. Overuse and misuse of antimicrobials for disease treatment and prevention have created a multifaceted problem as the number of multidrug resistant infections continues to rise at an average economic cost of $55 billion/year in the United States alone [1,2]. Traditionally, AMR research has focused on resistance originating from and circulating within human and animal populations, including transmission between the two, while neglecting the important role the environment plays in dissemination, transmission, and maintenance of antimicrobial resistant bacteria and genes [3,4]. However, the One Health approach to studying antimicrobial resistance aims to rectify this oversight by monitoring human, animal, and environmental contributions to the spread of AMR with the understanding that the health and functioning of each of these groups impact the others [5].

The spread of AMR between compartments can occur at multiple points of transmission but most frequently arises with contact of human and animal fecal matter [5,6]. Agricultural regions, especially concentrated livestock farms, are prime locations for transmission of AMR between humans, animals, and the environment due to the frequent use of manure for cropland fertilizer [7,8]. Manure has been cited as a “hot spot” for AMR proliferation and dissemination due to the frequent use of antimicrobials in livestock farming. In 2018, more than 11.5 million kg of antimicrobial drugs were used in food-producing animals in the United States including more than 31,000 kg of cephalosporin antibiotics, which are of critical importance for human health [9,10]. In such agricultural regions, animal manure can be considered the link between animal, human, and environmental AMR transmission due to the circuitous route of manure deposition on cropland for fertilization, runoff of manure into surface and ground waters, and human contact with either the livestock themselves or manure bacteria in recreational or drinking water. A better understanding of the dissemination and transmission of AMR between these three compartments, especially in areas of concentrated livestock farming, is critical to resistance mitigation.

*Escherichia coli*, a fecal indicator bacterium, is widely studied for the transmission of AMR between and within different sources as it is both an abundant intestinal bacterium found in most mammals as well as capable of surviving in natural environments [11]. The huge amount of genetic diversity within *E. coli* strains that has arisen due to evolution combined with the ability to survive in varied ecological niches make them the ideal bacterium to study AMR as they can be differentiated based on isolation source and genetic background [12,13]. Antimicrobial resistant *E. coli* have frequently been isolated from concentrated livestock farm animals as well as the surrounding environment but are rarely associated with both one another and clinical isolates within the same geographic region [14,15,16]. Similarities in AMR phenotypes, genetic determinants, and transmission vectors between *E. coli* isolates from humans, animals, and natural environments can help to identify hot spots of resistance as well as possible points for mitigation.

In this study, our primary aim was to identify associations between *E. coli* isolates collected from composite manure, clinical isolates, and freshwater ecosystems within the same geographic region and timeframe. By analyzing the isolates for a variety of phenotypic and genetic traits, including isolate phylotype, we characterized commonalities across isolates and identified patterns in AMR. Additionally, we targeted extended spectrum beta-lactamase (ESBL) producing *E. coli* and identified the genetic basis of ESBL production as well as transmission vectors.

## 2. Materials and Methods

### 2.1. Sample Collection and E. coli Isolation

Manure and environmental samples were collected in Kewaunee County, Wisconsin between 2017 and 2018, and clinical isolates were collected from hospitals serving the Kewaunee County community in Green Bay and Manitowoc, Wisconsin between 2017 and 2018. Kewaunee County, Wisconsin is home to sixteen concentrated animal feeding operations, primarily dairy farms, and is comprised of three primary river watersheds; a detailed description of the study area can be found in Beattie et al. [17]. Samples were collected and isolated as follows.

#### 2.1.1. Environmental Samples

Sediment and surface water grab samples were collected from the three primary river systems in Kewaunee County on multiple dates in 2017–2018, stored at 4 °C, and processed within 24 h (surface water) or 72 h (sediment). Approximately 1 g of homogenized sediment from each sample was mixed with 9 mL of sterile phosphate buffered saline and shaken at 200 rpm for 1 h. Serial dilutions of the mixture were plated in triplicate on modified membrane Thermotolerant *E. coli* agar (BD Difco™, Franklin Lakes, NJ, USA) plates and incubated at 37 °C for 2 h followed by 44.5 °C for 22 h. Magenta colonies were isolated as presumptive *E. coli*, streaked onto tryptic soy agar (Thermo Fisher™ Remel™, Lenexa, KS, USA), and further verified using the standard biochemical catalase (positive) and oxidase test (negative). Following verification, *E. coli* isolates were stored at −80 °C in tryptic soy broth (Thermo Fisher™ Remel™, Lenexa, KS, USA) with 25% glycerol (Sigma-Aldrich, Saint Louis, MO, USA) for downstream analyses. Surface water samples were filtered directly in varying volumes through 0.22 µm filters (MilliporeSigma Whatman™ Nuclepore, Burlington, MA, USA) city, state abbreviation, country. The filters were placed upright on mTEC agar and incubated, and *E. coli* were isolated, verified, and stored as above. A total of 64 environmental *E. coli* isolates were selected for use in this study.

#### 2.1.2. Manure Samples

Composite manure samples (contribution from >20 cows/sample) were collected from Kewaunee County cattle farms with owner permission on multiple dates in 2017–2018, stored at 4 °C, and homogenized and processed for *E. coli* isolation as in Section 2.1.1. for sediment above. A total of 65 manure *E. coli* isolates were selected for use in this study.

#### 2.1.3. Clinical Samples

Three study sites in Northeast Wisconsin were requested to annually submit 17 or 18 consecutive isolates of *Escherichia coli* identified from in-house routine microbiologic culture of clinically significant infection. Duplicate isolates (i.e., multiple isolates from the same patient course of illness from similar or different anatomical sources) were excluded. Isolates were maintained on Amies transport swabs containing charcoal (HealthLink, Jacksonville, FL, USA) prior to transport to Marquette University. Because of the lack of direct involvement in the collection of specimens and because of the utilization of de-identified isolates from routine clinical care, the Surveillance of Wisconsin Organisms for Trends in Antimicrobial Resistance and Epidemiology (SWOTARE) program was not considered to be actively engaged in human subject research by the Marquette University Institutional Review Board. Additional activities relative to the SWOTARE program have been described [18,19,20,21].

### 2.2. Antimicrobial Susceptibility Testing

Clinical, environmental, and manure *E. coli* isolates were subcultured twice for purity using trypticase soy agar with 5% sheep blood (ThermoFisher Scientific, Lenexa, KS, USA). Reference broth microdilution antimicrobial susceptibility testing was executed [22] and interpreted [23] using standards published by the Clinical and Laboratory Standards Institute (CLSI). In brief, turbidity of individual isolate suspensions was adjusted to a 0.5 McFarland standard and subsequently diluted 1:30 in Sensititre™ demineralized water (TREK Diagnostic Systems, East Grinstead, UK). Then, 95 pin polystyrene inoculator assemblies (Evergreen Scientific, Los Angeles, CA, USA) were filled with diluted contents for the subsequent inoculation of frozen Sensititre™ panels (TREK Diagnostic Systems, Cleveland, OH, USA) based on cation-adjusted Mueller–Hinton broth (Thermo Fisher™ Remel™, Lenexa, KS, USA). Panels consisted of customized dilution ranges that extended beyond CLSI breakpoints for the following antimicrobials: levofloxacin, ciprofloxacin, cefazolin, cefoxitin, ceftazidime, ceftriaxone, cefepime, ertapenem, meropenem, aztreonam, ampicillin, ampicillin-sulbactam, piperacillin-tazobactam, gentamicin, tobramycin, nitrofurantoin, and trimethoprim-sulfamethoxazole. Computerized audits of all isolates tested generated percentage susceptible, intermediate (susceptible-dose dependent for Enterobacterales/cefepime), and resistant values, as well as median minimum inhibitory concentration (MIC50) and 90th percentile (MIC90) determinations. Isolates resistant to three or more classes of antibiotics are classified as multidrug resistant.

### 2.3. ESBL Phenotyping

*E. coli* isolates showing intermediate or full phenotypic resistance to any cephalosporin, penicillin, and/or carbapenem antibiotics were further screened for ESBL production using the double-disk method proposed by the CLSI. Briefly, the disk diffusion method was used to screen isolate resistance to cefotaxime and ceftazidime both alone and in combination with 4 µg/mL clavulanic acid. An increase of ≥5 mm in zone diameter for either antimicrobial in combination with clavulanic acid compared to its zone diameter without clavulanic acid confirms the isolate is an ESBL producer. Antimicrobial disks alone and in combination with clavulanic acid were BD BBL™ Sensi-Disc™ brand (Franklin Lakes, NJ, USA). A total of 62 isolates (40 clinical, 15 environmental, and 7 manure) met the criteria to be screened for ESBL production.

### 2.4. ESBL and intI1 Gene Detection

DNA was extracted from all isolates using the Promega Wizard^®^ Genomic DNA Purification Kit (Madison, WI, USA). Because the ESBL phenotyping method above does not detect all ESBL producers, we additionally screened the 62 potential ESBL isolates for three common β-lactamase resistance genes by PCR: *bla_CTX-M_*, *bla_SHV_*, and *bla_TEM_.* Each simplex PCR reaction contained 12.5 µL of NEB Taq 2x master mix, 2.5 µL of MgCl_2_ (*bla_SHV_* only), 2.5 µL of F/R primer mix at 10 µM per primer (*bla_SHV_* and *bla_CTX-M_*) or 20 µM per primer (*bla_TEM_*), 5 µL of genomic DNA (2–50 ng/µL), and sterile water to 25 µL. Primers for each gene were as in Monstein et al. [24]. All PCR reactions were performed on a BioRad T100™ thermocycler (Hercules, CA, USA) with the following cycling parameters: 95 °C for 5 min followed by 30 cycles of 95 °C for 30 s, 60 °C for 30 s, and 70 °C for 1.5 min with a final cycle of 70 °C for 5 min. ESBL resistance gene controls were obtained from Dr. Christine Schneider (Carroll University, Waukesha, WI, USA). PCR products were verified via agarose gel electrophoresis (1.5% gels) and a portion of samples was subsequently sequenced. BLAST was used to identify the closest sequence match of each sequenced PCR product.

All isolates were additionally screened for the integron gene *intI1*, which has been identified as a marker of anthropogenic contamination and mobile transmission of antibiotic resistance genes (ARGs) [25]. Primers are as described in Beattie et al. [17]. Each reaction contained 12.5 µL of NEB Taq 2x master mix, 2.5 µL of F/R primer mix at 10 µM per primer, 5 µL of genomic DNA (2–50 ng/µL), and sterile water to 25 µL. All PCR reactions were performed on a BioRad T100™ thermocycler with the following cycling parameters: 95 °C for 10 min followed by 30 cycles of 95 °C for 60 s, 60 °C for 60 s, and 72 °C for 1.5 min with a final cycle of 72 °C for 10 min. Positive controls were obtained from *E. coli* isolates containing the verified cloned gene of interest. PCR products were verified via agarose gel electrophoresis (1% gels). 

### 2.5. Plasmid Detection and Plasmid Replicon Typing

The presence of plasmids in isolates that were ESBL positive (phenotype and/or genotype) was confirmed using the Promega Wizard^®^ *Plus* SV Miniprep DNA Purification kit (Madison, WI, USA) followed by agarose gel electrophoresis (1.5%) for plasmid DNA visualization. Plasmid positive isolates were subsequently replicon typed using the multiplex PCR method by Johnson et al. [26], which is a modified version of the one by Carattoli et al. [27]. Primers and PCR conditions can be found in Johnson et al. [26].

### 2.6. Phylogenetic Typing of E. coli Isolates

The quadruplex PCR method for *E. coli* phylogenetic relatedness from Clermont et al. [28] was used to assign all isolates in this study to one of the eight phylo groups. Primers and PCR cycling conditions, including the subsequent simplex PCR conditions for phylogroups A,C,D, and E, can be found in Clermont et al. [28].

### 2.7. Biofilm Formation Capability and Strength

The ability of each *E. coli* isolate to form biofilm was assessed using a crystal violet assay. Isolates were cultured overnight in Tryptic Soy Broth (TSB), diluted to 1 × 10^9^ CFU/mL, and a total of 200 µL was pipetted in triplicate into sterile 96 well plates (Nunc™ MicroWell™ 96-well Microplates, Waltham, MA, USA). Plates were incubated overnight for 24 h at 37 °C shaking at 150 rpm. Following overnight plate incubation, culture media was removed via pipette and plates were rinsed thrice with sterile phosphate buffered saline and allowed to air dry. Once dry, a total of 100 µL of 0.1% crystal violet solution was added to each well and plates were stained for 15 min at room temperature. After 15 min, the plates were rinsed thrice with sterile DI water and allowed to dry overnight. After drying, a total of 200 µL of 30% acetic acid was added to each well for 15 min to solubilize the biofilm and plate absorbance was read at 595 nm wavelength. Triplicate wells were averaged for absorbance. Biofilm formers were classified as weak (1 standard deviation above the media control), intermediate (2 standard deviations above the media control), or strong (3 standard deviations or more above the media control). Controls included *E. coli* strain ATCC 8739 (biofilm positive) and sterile culture media (negative).

### 2.8. Statistical Analyses

Categorical measured traits including antimicrobial resistance phenotype, gene presence, ESBL phenotype, and plasmid presence were converted into numerical code with 1 indicating presence and 0 indicating absence for binary variables and 0 indicating susceptible, 1 indicating intermediate, and 2 indicating resistant for antimicrobial resistance phenotype. Continuous variables (biofilm formation strength) were left unaltered. Principal coordinate analysis (PCoA) and permutation MANOVA followed by multiple pairwise *t*-tests were performed using the statistical software PRIMER-E (v7, PRIMER-E Ltd., Devon, United Kingdom) on a Gower distance matrix for mixed variables [29]. Proportional *Z*-test was used to identify significant differences between count data including percentages of antimicrobial resistance, genes, and phylotypes by isolate source. The multiple antibiotic resistance (MAR) index was calculated for each strain by taking the number of antibiotics to which an isolate was resistant and dividing it by the total number of antibiotics tested. MAR indices above 0.2 indicates that an isolate originated from an environment with elevated antibiotic use or a high risk source [30].

## 3. Results

### 3.1. Antimicrobial Resistance Phenotypes of Environmental, Cattle Manure, and Clinical E. coli Isolates

Isolates of *E. coli* collected in 2017–2018 from environmental samples (sediment and surface water) and cattle manure in Kewaunee County and clinical infections in hospitals serving the Kewaunee County region were analyzed for multiple traits including susceptibility to 17 antimicrobials, production of ESBLs or possession of ESBL genes, presence of plasmids and replicon types, biofilm formation capability, and phylogenetic group membership. The percentage of isolates resistant to at least one antimicrobial was highest in clinical isolates (47.5%), followed by environmental isolates (15.6%) and manure isolates (7.7%). Clinical isolates displaying resistance were, on average, resistant to two antimicrobials, with a range of isolate resistances from 0 to 11 antimicrobials. Although manure contained the fewest isolates displaying phenotypic resistance, the five isolates that did display phenotypic antimicrobial resistance were resistant to a minimum of six antimicrobials and up to a total of nine. While more environmental isolates were resistant to at least one antimicrobial than manure isolates, the maximum number of resistances for any one isolate was to five tested antimicrobials.

**Figure 1:**
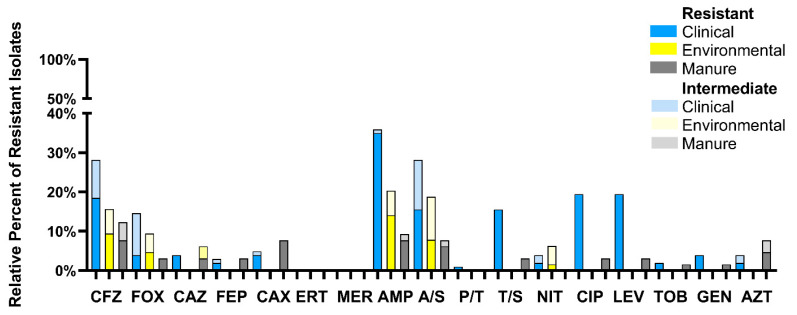
Percentage of resistant and intermediate resistant *Escherichia coli* isolates from clinical (blue), environmental (yellow), and manure (grey) environments. Antibiotic abbreviations: CFZ = cefazolin, FOX = cefoxitin, CAZ = ceftazidime, FEP = cefepime, CAX = ceftriaxone, ERT = ertapenem, MER = meropenem, AMP = ampicillin, A/S = ampicillin-sulbactam, P/T = piperacillin-tazobactam, T/S = trimethoprim-sulfamethoxazole, NIT = nitrofurantoin, CIP = ciprofloxacin, LEV = levofloxacin, TOB = tobramycin, GEN = gentamicin, AZT = aztreonam.

**Figure 2:**
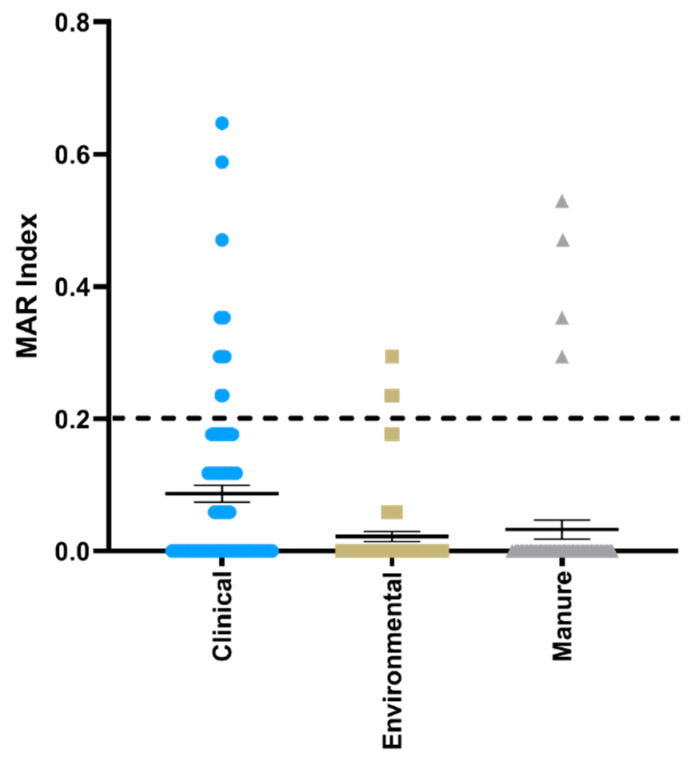
Multiple antibiotic resistance index (MAR) of clinical, environmental, and manure *E. coli* isolates. Isolates are presented as individual values with the mean and standard error of each source plotted. MAR indices >0.2 indicate that isolates likely originate from areas of high antibiotic use or a high risk source [30].

The relative percentage of isolates from each source resistant or intermediate resistant to the 17 tested antimicrobials is shown in Figure 1. Clinical isolates showed resistance to 15 of the tested antimicrobials, whereas manure isolates were resistant to 13 and environmental isolates were resistant to five. Clinical isolates were most resistant to ampicillin followed by ciprofloxacin, levofloxacin, and cefazolin, whereas manure isolates were most resistant to ampicillin, cefazolin, and ceftriaxone. Environmental isolates were most resistant to ampicillin followed by cefazolin, ampicillin-sulbactam, and cefoxitin. The multiple antimicrobial resistance index (MAR) of isolates by source is shown in Figure 2. The number of isolates with MAR indices over 0.2 from each source was 12 (11.7%), 3 (4.7%), and 5 (7.7%) for clinical, environmental, and manure isolates, respectively. 

Significant differences (*p* < 0.05) in the number of isolates from each isolation source identified as resistant to each antimicrobial were calculated using the proportional *Z*-test. Clinical isolates were significantly more resistant to ceftazidime, ceftriaxone, ampicillin, trimethoprim-sulfamethoxazole, ciprofloxacin, levofloxacin, and gentamicin than environmental isolates and significantly more resistant to cefazolin, ampicillin, trimethoprim-sulfamethoxazole, ciprofloxacin, and levofloxacin than manure. Environmental isolates were significant more resistant to ampicillin than manure isolates, whereas manure isolates were significantly more resistant to ceftazidime, ceftriaxone, trimethoprim-sulfamethoxazole, ciprofloxacin, and levofloxacin than environmental isolates and significantly more resistant to ceftriaxone and aztreonam than clinical isolates (Table 1).

### 3.2. Multiple Isolates from Each Source Were Phenotypically and/or Genotypically ESBL Positive

Isolates showing intermediate or full phenotypic resistance to any of the cephalosporin or penicillin antimicrobials were further screened for phenotypic and genotypic ESBL production using a disk diffusion assay and PCR for three ESBL genes (*bla_CTX-M_*, *bla_SHV_*, *bla_TEM_*). A total of 62 isolates (40 clinical, 15 environmental, and 7 manure) met the above criteria to be screened for ESBL production, with 35 of the 62 screened isolates (56.4%) identified as ESBL positive (15% of all study isolates). Seven isolates were phenotypic ESBL producers including four clinical isolates and three manure isolates. Of these seven isolates, three isolates contained at least one of the three screened ESBL genes including one manure isolate with both *bla_CTX-M_* and *bla_TEM_*, one manure isolate with *bla_CTX-M_* only, and one clinical isolate with *bla_TEM_* only. An additional 28 isolates that were not phenotypic ESBL producers were found to harbor at least one of three tested ESBL genes and included 23 clinical isolates, 2 manure isolates, and 3 environmental isolates (Table 2). Of these, one clinical isolate contained both *bla_CTX-M_* and *bla_TEM_* genes, one clinical isolate contained only *bla_CTX-M_*, and the remaining 26 isolates contained only *bla_TEM_.* No isolate from this study contained *bla_SHV_*. Sequencing of the ESBL genes revealed that the two *bla_CTX-M_* genes harbored by clinical isolates were most homogenous with *bla_CTX-M-15_*, whereas *bla_CTX-M_* from manure was most homogenous with *bla_CTX-M-161_*. Overwhelmingly, the *bla_TEM_* genes shared the most sequence homology with *bla_TEM-1_*, although one *bla_TEM-29_* and one *bla_TEM-105_* were also identified in two separate clinical isolates.

Clinical isolates were significantly more likely to be ESBL positive compared to environmental isolates (*p* = 0.0022) and manure isolates (*p* = 0.0315) and were also significantly more likely to contain the *bla_TEM_* gene than environmental isolates (*p* = 0.0096, Table 1). Six of the seven isolates (85.7%) that displayed phenotypic ESBL production had MAR indices >0.2, and five of the seven had MAR indices >0.45. Isolates containing ESBL genes without phenotypic resistance were less likely to have elevated MAR indices than phenotypic ESBL producers with only eight of the 28 (28.6%) gene positive isolates having MAR indices > 0.2 (Table 2). Clinical and manure isolates that were ESBL positive were commonly found to be co-resistant to quinolone antimicrobials (ciprofloxacin, levofloxacin) and aminoglycoside antimicrobials (gentamicin). Environmental isolates that were ESBL positive only showed co-resistance to ampicillin (Table 2).

### 3.3. Presence of Mobile Genetic Element Indicators, Plasmids, and Plasmid Replicon Type

All study isolates were screened for the integrase gene *intI1* that contributes to the mobility of antimicrobial resistance genes found on integrons. Surprisingly, only four clinical isolates, one environmental isolate, and zero manure isolates contained this gene; of these, only one isolate (clinical) was also ESBL positive (*bla_TEM_*) (Table 2).

The horizontal gene transfer of antimicrobial resistance including ESBL genes between *E. coli* isolates is a common, and concerning, occurrence; thus, all 62 presumptive ESBL positive isolates were also screened for the presence of plasmids. Of those screened, 15 contained plasmids as identified by agarose gel electrophoresis and plasmid typing, including 11 clinical isolates, three manure isolates, and one environmental isolate, 12 of which were also ESBL positive (Table 2). Nine plasmid replicon types were identified in clinical samples, with the most prevalent being IncFIA (45.5%) followed by IncFIC > IncFIB = IncA/C = IncI1 > IncT = IncP = IncN. Two of the three manure isolates had identifiable plasmid replicon types and were nearly identical with both containing IncB/O, IncFIA, and IncFIB. One manure isolate also contained IncI1, whereas the other isolate contained IncK/B. The single environmental isolate contained only one plasmid replicon type, namely IncFIC. Isolates containing IncFIA, regardless of isolation source, were more likely to be co-resistant to levofloxacin, ciprofloxacin, and/or gentamycin and have MAR indices >0.2 (Table 2).

### 3.4. Biofilm Formation Capability and Correlation with Antimicrobial Resistance Phenotype

**Figure 3:**
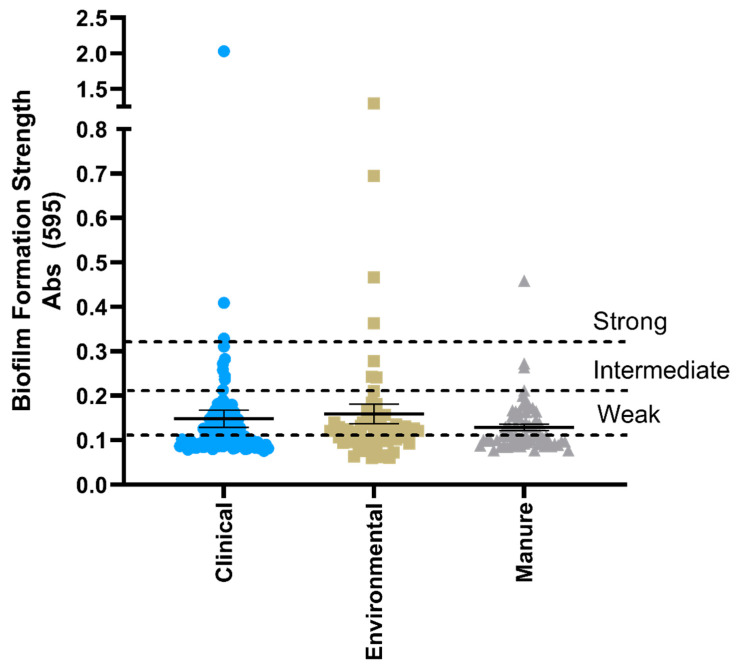
Biofilm formation strength of *E. coli* isolates as measured by absorbance of crystal violet staining. Isolates are presented as individual values with the mean and standard error of each source plotted. Strong, intermediate, and weak biofilm former classification is defined in Section 2.7, Materials and Methods.

Biofilm formation is an important component of bacterial survival and increases the ability of a bacterium to resist or tolerate antimicrobial treatment. Isolates, regardless of isolation source, were equally likely to form measurable biofilm, with a total of 41 clinical (39.8%), 26 environmental (40.1%), and 26 manure (40.0%) isolates forming biofilm at varying strengths (Figure 3). Although biofilm formation strength was frequently found in conjunction with plasmid positive and multiple resistant isolates, the ability of an isolate to form a biofilm was equally as likely to occur without any phenotypic or genotypic resistances (Table 2), indicating that this life form is common in both pathogenic and commensal *E. coli* isolates.

### 3.5. Associations between Isolate Phylogenetic Group, Antimicrobial Resistance Profile, and Isolation Source

**Figure 4:**
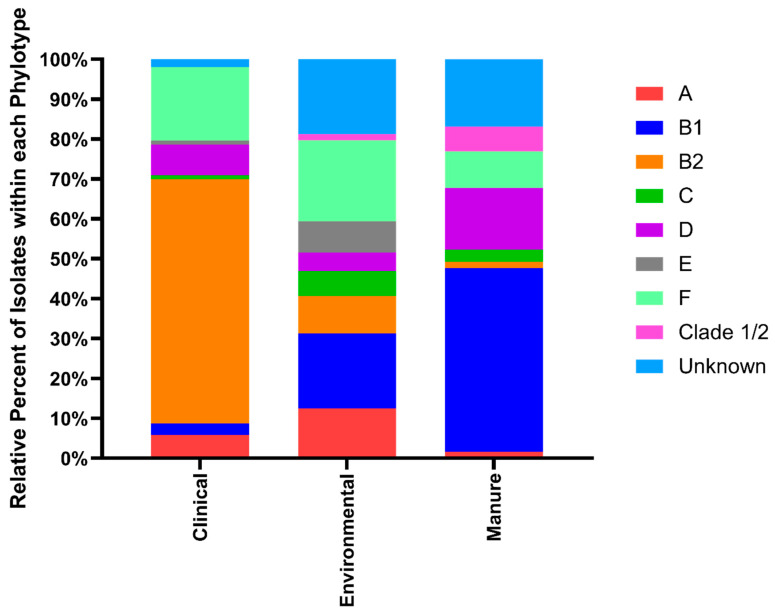
Relative percentage of *E. coli* isolate phylotype within clinical, environmental, and manure isolation sources.

*E. coli* bacterial strains contain significant genetic substructure and can be classified into phylogroups based on evolutionary relatedness. In general, different phylogroups of *E. coli* are not randomly distributed and thus phylotyping isolates from different sources can help identify transmission between and intermixing of *E. coli* from different isolation sources. Using the well-accepted *E. coli* phylotyping method [28], we classified all strains in this study into one of eight phylogroups including B2 and D, which are commonly pathogenic strains, A and B1, which are common commensal strains, and C, E, F, and Clade 1, the less common but identifiable *E. coli* phylotypes. The breakdown of phylotype groupings by isolate source can be seen in Figure 4. Clinical isolates were overwhelmingly typed to the B2 group with 61.2% falling within this phylotype, whereas environmental isolates were primarily typed to B1 (18.8%) or F (20.3%), and manure isolates were primarily typed to B1 (46.2%) or D (15.4%). A limited number (10.8%) of isolates in this study were not assigned to a definitive phylotype, which is common in isolates from mixed sources as in this study.

**Figure 5:**
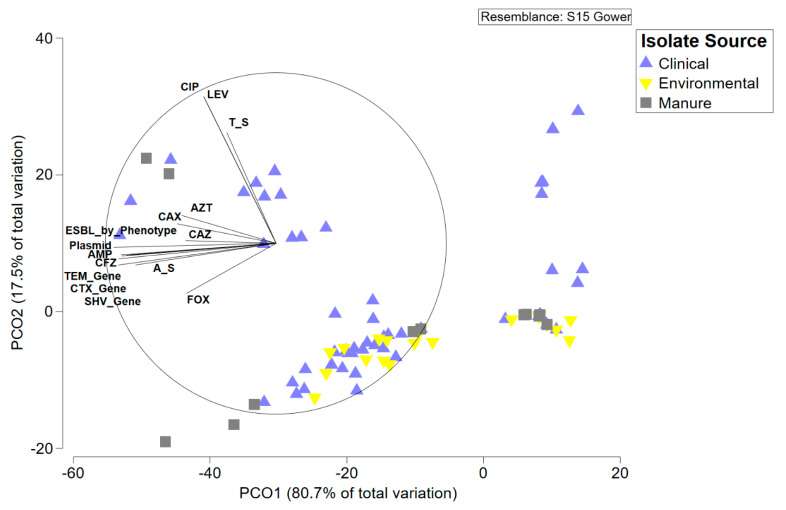
Principal coordinate analysis of all measured traits of *E. coli* isolates in this study based on Gower distance. Measured traits with a correlation strength of >0.5 are displayed on the plot.

The primary goal of this study was to identify associations between *E. coli* isolated from different sources within a localized geographic area based on genetic background, antimicrobial resistance profile, and other measured traits. Principal coordinate analysis was used to explore these differences in *E. coli* profiles from the three isolation sources (Figure 5). Environmental and manure isolates primarily clustered together and were negatively correlated with presence of ESBL genes and resistance to multiple antimicrobials. Clinical isolates were more widely distributed with a cluster of susceptible isolates co-located with the environmental–manure cluster and a cluster of resistant and ESBL isolates that strongly correlated with the associated variables (Figure 5). To determine if these measured variables created significant differences between isolate source and/or isolate phylotype, we used permutated MANOVA followed by subsequent post-hoc *t*-tests. *E. coli* isolates were significantly different based on isolation source and isolate phylotype (Table 2). Subsequent post-hoc *t*-tests indicated that clinical isolates were significantly different from manure isolates and near significant difference from environmental isolates due to their overrepresentation in the B2 phylogroup. Environmental and manure isolates were not significantly different by isolation source or phylogroup.

## 4. Discussion

The One Health approach to antimicrobial resistance aims to understand the intersection of resistance between humans, animals, and the environment to better mitigate the spread of resistant bacterial infections. In this study, our goal was to assess the phenotypic and genotypic similarities of *E. coli* isolates collected from manure, freshwater ecosystems, and clinical samples within a vulnerable community to determine associations between isolate source and possible mechanisms of resistance transmission. We found that isolates from manure and environmental sources were more phenotypically and genotypically similar to one another than clinical isolates from the same region, which is consistent with reports of widescale manure contamination of freshwater sources in the county [17,31]. Although manure and environmental isolates were less likely to display phenotypic antimicrobial resistance or to produce ESBLs than clinical isolates, they were equally as likely to form biofilm and harbor plasmids that are known to contain multiple antimicrobial resistance genes [32]. Clinical isolates were resistant to the greatest number of antimicrobials and were overwhelmingly assigned to the phylotype B2, which is associated with pathogenic *E. coli* strains [26,33], similar to other studies [13]. However, several environmental and manure isolates were also typed to both the B2 phylotype and D phylotype, which is also considered pathogenic [26], indicating that a portion of these isolates likely harbor virulence markers. Phylotypes of manure and environmental isolates displayed more evenness than clinical isolates that were dominated by the B2 phylotype. No significant differences in phylotype or isolation source based on measured traits were identified between manure and environmental isolates, which is likely due to frequent intermixing of these two environments in Kewaunee County.

*E. coli* isolates across source environments in this study were most frequently resistant to ampicillin, followed by ampicillin-sulbactam, cefazolin, and cefoxitin. This result is concerning as these antibiotics are classified as highly important or critically important for human medicine [9]. Biofilm formation capability is an important component of clinical bacterial infections and is frequently associated with antibiotic tolerance; thus, we assumed clinical isolates would have a higher biofilm formation strength. Interestingly, no differences in the biofilm formation ability of the isolates were identified between source environment as approximately 40% of isolates from each source could form detectable biofilm (Figure 3). However, the genetic diversity and plasticity of *E. coli* suggests that biofilm formation would benefit isolates across source type and aid in protection against physical and chemical stressors [12,34].

Understanding the characteristics of *E. coli* capable of producing ESBLs is of critical importance for human and veterinary health [35,36]. While we only identified seven phenotypic ESBL producer isolates in this study, an additional 28 isolates were found to harbor ESBL resistance genes. Other studies of Gram-negative bacteria have identified similar false-negative issues with ESBL phenotyping [37,38]. A significant number (51.4%) of ESBL positive isolates were classified to the B2 phylotype and 40% had MAR indices of >0.2. This indicates that these isolates are both resistant to antimicrobials of last resort and many are multidrug resistant. Other reports also find that ESBL isolates are frequently typed to the B2 phylotype and harbor virulence factors [39,40]. The primary ESBL gene identified across all isolates was *bla_TEM-1_*, corroborating other studies where this gene was also the most common *bla* gene in Gram-negative ESBL producers [41,42]. Additionally, *bla_TEM-1_* is plasmid-mediated [41,43], thus horizontal transfer of this gene has the capability to occur between isolates of all three source types. Together, these data indicate that isolate phylotype, regardless of isolation environment, may play an important role in strain AMR determination, indicating phylotype of *E. coli* strain should be more closely monitored.

Isolates from all three sources harbored plasmids, with nine replicon types found in clinical isolates, five in manure isolates, and one in an environmental isolate. While some plasmid replicon types were shared between clinical and manure isolates, including IncFIA, IncFIB, and IncI1, and between clinical and environmental isolates, including IncFIC, the phenotypic and genotypic resistance patterns were not identical, indicating that the pool of mobile genetic elements within different isolate sources from Kewaunee County is diverse. It is important to note that isolates containing plasmid replicon type IncFIA were more likely to be ESBL producers and co-resistant to ciprofloxacin, levofloxacin, and gentamicin with MAR indices >0.2 (Table 2). Other recent studies including Ali et al. [44] and Fagerstrom et al. [45] also found that clinical ESBL-producing isolates were most likely to harbor IncFIA-FIB replicon types. It is interesting to note that the manure ESBL isolates also frequently contained IncFIA-FIB replicon types as the clinical isolates, but additional sequence similarity information is needed to fully understand the relationship and association between the plasmids from different sources.

Isolates from clinical samples were significantly different based on the multiple traits measured in this study and, as a whole, were significantly more likely to be classified to the B2 phylogroup based on permutational MANOVA results (Table S1, Supplementary Materials). Interestingly, isolate source and isolate phylotype of manure and environmental *E. coli* were not significantly different from one another based on measured traits. This suggests that manure and environmental *E. coli* isolates in this study are more closely related to one another than to clinical isolates and is indicative of the well-known manure contamination of freshwater sources in Kewaunee County [17,31]. Other studies have shown that animal feces can transmit AMR *E. coli* to freshwater sources, where the bacterium can survive, proliferate, and continue to spread AMR [46,47]. This suggests that the One Health approach to AMR surveillance is necessary to detect transmission between various source environments.

In this study, we identified a strong link between manure and freshwater *E. coli* including similar phylotypes, plasmid replicon types, and AMR phenotypes. Although overall resistance was lower in these isolates compared to clinical isolates, the spread of *E. coli* that may harbor resistance determinants poses a serious risk to human health, especially for those who come into contact with or drink from contaminated waterways. Manure isolates in this study displaying resistance are a particular concern because, although few displayed phenotypic resistances, those that did were resistant to a minimum of six antibiotics. Widespread runoff of manure used as cropland fertilizer increases the risk of highly resistant isolates entering into surface and groundwater systems, putting public health at risk. Future work should include sequencing of the plasmids identified in this study to assess similarity across sampling sources and identify possible horizontal gene transfer occurrences. Care should be used when undertaking the difficult task of assessing the transfer and association of isolates from different sources even when collected within the same geographic location and timeframe.

## Figures and Tables

**Table 1:** Significant differences in resistance determinant (phenotypic resistance, gene presence, and extended spectrum beta-lactamase (ESBL) production) between isolate source based on proportional *Z*-test.

Resistance Determinant	Groups	*p*-Value
CFZ	Clinical > Manure	0.04478
CAZ	Clinical > Environmental Manure > Environmental	0.01882 0.009802
FEP	Manure > Environmental	0.009802
CAX	Clinical > Environmental Manure > Clinical, Environmental	0.01882 0.001357, <0.0001
AMP	Clinical > Environmental, Manure Manure > Environmental	0.000182, <0.0001 0.005195
T/S	Clinical > Environmental, Manure Manure > Environmental	<0.0001, 0.000512 0.009533
CIP	Clinical > Environmental, Manure Manure > Environmental	<0.0001, <0.0001 0.009566
LEV	Clinical > Environmental, Manure Manure > Environmental	<0.0001, <0.0001 0.009566
GEN	Clinical > Environmental	0.0188
AZT	Manure > Clinical	0.0085
TEM gene +	Clinical > Environmental	0.009644
ESBL producer	Clinical > Environmental Manure > Environmental	0.002208 0.03145

**Table 2:** Analyzed traits for *E. coli* isolates that were phenotype or genotypic ESBL producers or plasmid positive.

Isolate ID	Source Environment	Resistance Phenotype	MAR Index	ESBL by Phenotype	*bla_CTX_* Gene Positive	*bla_TEM_* Gene Positive	*intI1* Gene Positive	Plasmid Presence	Plasmid Replicon Type	Phylotype	Biofilm Formation Strength
1275	Clinical	CFZ-CAZ-FEP-CAX-AMP-A/S-CIP-AZT-TOB-GEN-LEV	0.6471	Yes	No	No	No	No		B2	0.090
1276	Clinical	CFZ	0.0588	Yes	No	No	No	Yes	FIC, I1	D	0.119
1842	Clinical	CFZ-FOX-CAZ-FEP-CAX-AMP-A/S-CIP-AZT-LEV	0.5882	Yes	No	No	No	Yes	FIA	D	0.099
2561	Clinical	CFZ-CAZ-AMP-CIP-TOB-GEN-LEV-T/S	0.4706	Yes	No	TEM-29	No	Yes	FIA	B2	0.091
1269	Clinical	AMP-A/S	0.1176	No	No	TEM-1	No	No		B2	0.093
1270	Clinical	CFZ-AMP-A/S-CIP-LEV	0.2941	No	No	TEM-105	No	No		B2	0.164
1273	Clinical	CFZ-P/T-AMP-A/S-	0.2353	No	No	TEM-1	No	Yes	A/C, T, I1, B/O	B2	0.311
1309	Clinical	AMP	0.0588	No	No	Present, not sequenced	No	No		B2	0.092
1841	Clinical	AMP	0.0588	No	No	TEM-1	No	No		B2	0.096
1843	Clinical	AMP	0.0588	No	No	Present, not sequenced	No	Yes	B/O	B1	0.128
1846	Clinical	AMP-CIP-GEN-LEV-T/S	0.2941	No	No	TEM-1	No	Yes	P, FIC, FIA	B2	0.184
1848	Clinical	AMP-T/S	0.1176	No	No	TEM-1	No	Yes	A/C, FIC, FIA, FIB	F	0.102
1856	Clinical	AMP	0.0588	No	No	TEM-1	No	Yes	FIB	F	0.132
1857	Clinical	AMP-A/S	0.1176	No	No	Present, not sequenced	No	No		F	0.147
1895	Clinical	AMP	0.2353	No	No	Present, not sequenced	No	No		F	0.104
1896	Clinical	CFZ-AMP-A/S	0.1765	No	No	TEM-1	No	No		F	0.101
1921	Clinical	CFZ-FOX-AMP	0.1765	No	No	TEM-1	No	Yes	FIC, N	B2	0.109
2540	Clinical	CFZ-FOX (intermediate resistance only)	0.0000	No	No	TEM unclassified	Yes	No		F	0.167
2543	Clinical	CFZ-AMP-A/S	0.1765	No	CTX-M-15	TEM-1	No	No		B2	0.175
2558	Clinical	AMP-GEN-T/S	0.1765	No	No	Present, not sequenced	No	No		F	0.129
2564	Clinical	AMP-A/S	0.1176	No	No	TEM-1	No	No		B2	0.082
2603	Clinical	AMP-CIP-LEV	0.1765	No	No	Present, not sequenced	No	No		B2	0.162
2606	Clinical	AMP-A/S-CIP-LEV-T/S	0.2941	No	No	Present, not sequenced	No	No		B2	0.178
2616	Clinical	AMP-CIP-LEV	0.1765	No	No	TEM-1	No	Yes	A/C, FIA, I1	B2	0.079
2715	Clinical	AMP-A/S	0.1176	No	No	Present, not sequenced	No	No		B2	0.096
2600	Clinical	CFZ-AMP-CIP-LEV-T/S	0.2941	No	CTX-M-15	No	No	No		B2	0.127
2535	Clinical	CFZ-CAX-AMP-CIP-LEV-T/S	0.3594	No	No	TEM unclassified	No	No		B2	0.182
1318	Clinical	AMP-A/S	0.1176	No	No	No	No	Yes	B/O, FIB	B2	0.190
2017 M23	Environmental	AMP	0.0588	No	No	TEM-1	No	No		B1	0.106
M45	Environmental	CFZ	0.0588	No	No	TEM-1	No	No		unknown	0.131
M25	Environmental	AMP	0.0588	No	No	TEM-1	No	No		unknown	0.125
M69	Environmental	CFZ-AMP-A/S	0.1765	No	No	No	No	Yes	FIC	unknown	0.107
E2–4	Manure	CFZ-FEP-CAX-AMP-A/S-CIP-AZT-LEV-T/S	0.5294	Yes	Present, not sequenced	TEM-1	No	No		Clade 1/2	0.132
E1–10	Manure	CFZ-CAX-AMP-A/S-CIP-AZT-LEV-T/S	0.4706	Yes	No	No	No	Yes	N/A- no clear type	Clade 1/2	0.190
M6	Manure	CFZ-FEP-CAX-AMP-AZT	0.2941	Yes	CTX-M-161	No	No	No		unknown	0.459
2017 C52	Manure	CFZ (intermediate resistance only)	0.0000	No	No	TEM unclassified	No	No		B1	0.089
M9	Manure	CFZ-FOX-CAZ-CAX-AMP-A/S-TOB-GEN	0.4706	No	No	TEM-1	No	Yes	B/O, FIA, FIB, I1	B2	0.138
M8	Manure	CFZ-FOZ-CAZ-CAX-AMP-A/S	0.3529	No	No	No	No	Yes	B/O, FIA, FIB, K/B	Clade 1/2	0.264

## Supplementary Materials

The following are available online at https://www.mdpi.com/2076-2607/8/5/747/s1, Table S1. Comparison of Gower distance of *E. coli* isolates based on all analyzed traits by isolate source and phylotype using permuted MANOVA.
