# Supplementary material for: Antimicrobial Resistance Traits of Escherichia coli Isolated from Dairy Manure and Freshwater Ecosystems Are Similar to One Another but Differ from Associated Clinical Isolates
Source: Microorganisms. 2020 May 16;8(5):747. doi: 10.3390/microorganisms8050747 (PMC7284991; doi:10.3390/microorganisms8050747)
Supplement: Supplementary file 1 [file microorganisms-08-00747-s001.pdf]

**Table S1.** Comparison of Gower distance of *E. coli* isolates based on all analyzed traits by isolate source and phylotype using permuted MANOVA. Bolded values indicate significant differences ($p<0.05$).

| **MANOVA** | | |
|---|---|---|
| *Variable* | *Pseudo-F* | *P(perm)* |
| Isolate Source | 4.2451 | **0.012** |
| Isolate Phylotype | 2.6019 | **0.011** |
| Source x Phylotype | 2.0158 | **0.016** |
| **MANOVA Pairwise Tests- Isolate Source** | | |
| *Groups* | *t* | *P(perm)* |
| Clinical, Environmental | 2.8048 | **0.002** |
| Clinical, Manure | 2.7558 | **0.004** |
| Environmental, Manure | 0.41641 | 0.82 |
| **MANOVA Pairwise Tests- Phylotype B2** | | |
| *Groups* | *t* | *P(perm)* |
| Clinical, Environmental | 1.6119 | 0.087 |
| Clinical, Manure | 2.3328 | **0.042** |
| Environmental, Manure | 3.351 | 0.14 |
| **MANOVA Pairwise Tests- Phylotype D** | | |
| *Groups* | *t* | *P(perm)* |
| Clinical, Environmental | 0.83976 | 0.595 |
| Clinical, Manure | 1.6258 | 0.056 |
| Environmental, Manure | 1.043 | 0.5216 |
| **MANOVA Pairwise Tests- Phylotype E** | | |
| *Groups* | *t* | *P(perm)* |
| Clinical, Environmental | 2.0077 | 0.177 |
| **MANOVA Pairwise Tests-Phylotype B1** | | |
| *Groups* | *t* | *P(perm)* |
| Clinical, Environmental | 0.55627 | 0.676 |
| Clinical, Manure | 1.8977 | 0.095 |
| Environmental, Manure | 1.7169 | 0.089 |
| **MANOVA Pairwise Tests- Phylotype A** | | |
| *Groups* | *t* | *P(perm)* |
| Clinical, Environmental | 1.2185 | 0.215 |
| Clinical, Manure | 0.28313 | 1 |
| Environmental, Manure | 0.66569 | 0.88 |

| **MANOVA Pairwise Tests- Phylotype F** | | |
|---|---|---|
| *Groups* | *t* | *P(perm)* |
| Clinical, Environmental | 1.5916 | 0.104 |
| Clinical, Manure | 1.6472 | 0.128 |
| Environmental, Manure | 1.3304 | 0.214 |

| **MANOVA Pairwise Tests- Phylotype C** | | |
|---|---|---|
| *Groups* | *t* | *P(perm)* |
| Clinical, Environmental | 0.44721 | 1 |
| Environmental, Manure | 0.66667 | 1 |

| **MANOVA Pairwise Tests- Phylotype Clade 1/2** | | |
|---|---|---|
| *Groups* | *t* | *P(perm)* |
| Environmental, Manure | 1.1345 | 0.583 |
